# Cyberattack patterns in blockchain-based communication networks for distributed renewable energy systems: A study on big datasets

**DOI:** 10.1016/j.dib.2024.110212

**Published:** 2024-02-22

**Authors:** Muhammad Faheem, Mahmoud Ahmad Al-Khasawneh, Arfat Ahmad Khan, Syed Hamid Hussain Madni

**Affiliations:** a School of Computing Technology and Innovations, University of Vaasa, Vaasa 65200, Finland; b Vaasa Energy Business and Innovation Centre (VEBIC), University of Vaasa, Vaasa 65200, Finland; c School of Digital Economy, University of Vaasa, Vaasa 65200, Finland; d School of Computing, Skyline University College, University City Sharjah, Sharjah 1797, the United Arab Emirates; e Department of Computer Science, College of Computing, Khon Kaen University, Khon Kaen 40002, Thailand; f School of Electronics and Computer Science, University of Southampton Malaysia, Johor Bahru 79100, Malaysia

**Keywords:** Blockchain, Solana, Cybersecurity, Internet of things, Distributed energy systems, Smart grid

## Abstract

Blockchain-based reliable, resilient, and secure communication for Distributed Energy Resources (DERs) is essential in Smart Grid (SG). The Solana blockchain, due to its high stability, scalability, and throughput, along with low latency, is envisioned to enhance the reliability, resilience, and security of DERs in SGs. This paper presents big datasets focusing on SQL Injection, Spoofing, and Man-in-the-Middle (MitM) cyberattacks, which have been collected from Solana blockchain-based Industrial Wireless Sensor Networks (IWSNs) for events monitoring and control in DERs. The datasets provided include both raw (unprocessed) and refined (processed) data, which highlight distinct trends in cyberattacks in DERs. These distinctive patterns demonstrate problems like superfluous mass data generation, transmitting invalid packets, sending deceptive data packets, heavily using network bandwidth, rerouting, causing memory overflow, overheads, and creating high latency. These issues result in ineffective real-time events monitoring and control of DERs in SGs. The thorough nature of these datasets is expected to play a crucial role in identifying and mitigating a wide range of cyberattacks across different smart grid applications.

Specifications Table

**Table:** 

Subject	Computer Science: Computer Communication Networks, Distributed Energy Systems, Integration of Renewable Power Systems.
Specific subject area	Cybersecurity, Blockchain.
Data format	Raw and Analyzed
Type of data	Tables, Graphs, Figures
Data collection	Data were acquired from the Solana blockchain-based IWSNs positioned in geographically distributed wind turbines in a wind farm. The deployed sensor nodes sense and collect various types of events information and send this data to the head node called the sink in a multi-hop manner. The sink forwards the collected data in real-time to the data center using Internet of Things (IoT)-enabled wired or wireless communication technology for further investigations. An adversary A launches a set of cyber attacks including, SQL Injection, Spoofing, and Man-in-the-Middle to pose data leakage, malicious tampering, and identity validity threats on the distributed energy systems involved in the power generation, transmission, and distribution in the smart grid. The datasets of various types of under-attacked sensor nodes involved in events monitoring and control in the wind farm were collected thought the developed Smart Communication Framework (SCF) in DERs.
Data source location	Institution: University of Vaasa
	City/Town/Region: Palosaari, 65,200, Vaasa.
	Country: Finland.
	Latitude and longitude for collected samples/datasets: 63°06'13.6”N 21°35'36.4”E.
Data accessibility	Data are available in this article and at the Mendeley Data repository.
	Direct URL to data: https://data.mendeley.com/datasets/k2gj4pssyr/2
	Doi: 10.17632/k2gj4pssyr.2
Related research paper	Big datasets are novel and have not been published previously, are the part of our research work presented in reference [1].

## 1 Value of the Data

• The cybersecurity research community, especially those focusing on energy and power sectors, can derive significant value from these datasets in enhancing smart grid applications.
• These rarely made-available cybersecurity datasets allow researchers to effectively distinguish between normal and abnormal system behaviors in power generation, transmission, and distribution processes.
• Analysis of these datasets is instrumental in predicting the patterns of cyberattacks, including their frequency and continuity, particularly in distributed renewable energy systems. This knowledge is crucial for designing and developing advanced solutions for anomaly detection and mitigation in the power and energy sector.
• The collaboration between the cybersecurity and energy sectors, along with other stakeholders, is essential in utilizing these datasets to fortify communication infrastructures in the smart grid. This effort is essential for protecting the privacy of employees, organizations, and customers.
• Enhancing the datasets with expert-annotated semantics also improves their credibility, trustworthiness, and access control. In remote system applications, such as those in e-health, e-transportation, e-agriculture, and other fields, this enrichment is especially helpful. Such comprehensive utilization and enhancement of the datasets promise a more secure and resilient future in these fields.

## 2 Background

The needs for more energy is rising day by day, pushing electric power companies to instantly integrate green energy sources in the smart grid using advanced information and communication technologies (ICTs) [2], [3], [4]. However, the ICTs in DERs are susceptible to various kinds of cyberattacks such as SQL Injection, Spoofing, Man-in-the-Middle, cloning, and others [5], [6], [7], [8], [9]. Therefore, innovative solutions are essential and must be integrated to improve the resilience, stability, and efficiency of the DERs in the SG [10], [11], [12], [13]. The blockchain technology offers a reliable, resilient, and secure information exchange architecture for monitoring and control of DERs in SG [14], [15], [16], [17]. In this regard, some advanced blockchain technologies with different characteristics have been listed in Table 1 [18], [19], [20], [21] for various types of SG applications shown in Table 2 [22]. Consequently, this study presents big cybersecurity datasets for further analyses, interpretations, and visualizations that were not fully explored in the original research, thereby enriching the understanding of the framework's efficiency in energy and power systems security. The big datasets were collected from various wind turbines in a wind farm, reveal nuanced aspects of the cybersecurity framework, contributing to a more comprehensive view of its potential and limitations. By making this extensive data and methodological information available, the data article fosters further cybersecurity research and innovation in blockchain-based infrastructure in various energy and power systems applications.

**Table 1:** Blockchain technologies for IWSNs in smart grid applications.

Metrics	Bitcoin	Ethereum	Aptos	Solana	Palkadot	Avalanche
Type of blockchain	Layer 1	Layer 1	Layer 1	Layer 1	Layer 1	Layer 1
Architecture	Pub/Pvt	Pub/Pvt	Pub/Pvt	Pub/Pvt	Pub/Pvt	Pub/Pvt
Consensus mechanism	PoW	PoS	PoS	PoS and PoH	PoS	PoS
Maximum transaction per second	7+tps	45+tps	160,000	5000+tps	1500+tps	10,000+tps
Hash Function	SHA-256	Keccak-256	SHA-256	SHA-256	Blake2b	secp256k1
Time-To-Finality	60 min	> 5 min	< 1 s	<2.5 s	6 min	< 2 s
Number of Validators	Pools w/ > 51% hash rate	2 Pools w/ >51% hash rate	<102 nodes relay chain	Thousands of nodes	<200 nodes relay chain	Thousands of nodes
Safety Threshold	51%	51%	33%	66%	33%	80%
Programming language	C++	Solidity	Move	Rust, C, C++, Python	Rust to JavaScript	Go, TypeScript, JavaScript, Python, Vue
Implementation complexity	-	–	–	–	-	O(kn)
Latency	High	Moderate	Low	Low	Moderate	Low
Scalability	Low	Moderate	Moderate	High	High	High
Energy Efficiency	No	No	No	Yes	Yes	Yes

**Table 2:** Communication requirements for blockchain-based IWSNs in smart grid applications.

Sr.#	Applications	Security	Bandwidth	Reliability	Latency	Technology
1	Home energy management (HEM)	High	9.6–56 kbps	99.0–99.99%	up to 2 s	5 G (300 Mbps)/ Optical (1 Gbps)
2	Advanced metering infrastructure (AMI)	High	10–100 kbps per node, 500 kbps for backhaul	99.0–99.99%	<10 s	
2(a)	Meter reading – on-demand	High	100 bytes	>98%	<15 s	
2(b)	Meter reading – scheduled manner	High	1.6k-2.4 kbps	>98%	<4 h	
2(c)	Meter reading – collective manner	High	>=1000 kbps	99.0%	<1 h	
3	Wide-area situational awareness	High	600–1500 kbps	99.0%	up to 5 s	
4	Demand response management (DRM)	High	14–100 kbps per node	99.0%	up to several minutes	
5	Substation automation (SA)	High	9.6–56 kbps	99–100%	up to 1 s	
6	Outage management (OM)	High	56 kbps	99.0%	2 s	
7	Distribution management (DM)	High	9.6–100 kbps	99.0–99.99%	up to 5 s	
8	Distribution generation (DG)	High	9.6–56 kbps	99.0%	2 s	
9	SCADA	High	56–100 kbps	99.0%	<3 s	
10	Monitoring and Control (MC)	High	56–100 kbps	99.0–99.99%	<2 s	
11	Asset management (AM)	High	56 kbps	99.0%	<5 s	
12	Meter data management (MDM)	High	56 kbps	99.0%	<10 s	
13	Transmission line monitoring	High	9.6–64 kbps	90.0%	up to 5 s	
14	Distributed energy resources and storage (DERs)	High	9.6–56 kbps	99.0–99.99 %	up to 5 s	
15	Vehicle to grid (VG)	High	9.6–56 kbps	99.0–99.99%	2 s-5 min	
16	Electrical vehicles (EV)	High	9.6–56 kbps	99.0–99.99%	2 s-5 min	
17	Program/configuration update	High	25–50kbps	>98%	<5 min-7days	
18	Firmware update (FU)	High	400 kb/s-2000 kbps	>98%	<2 min-7days	

## 3 Data Description

**Fig. 1:**
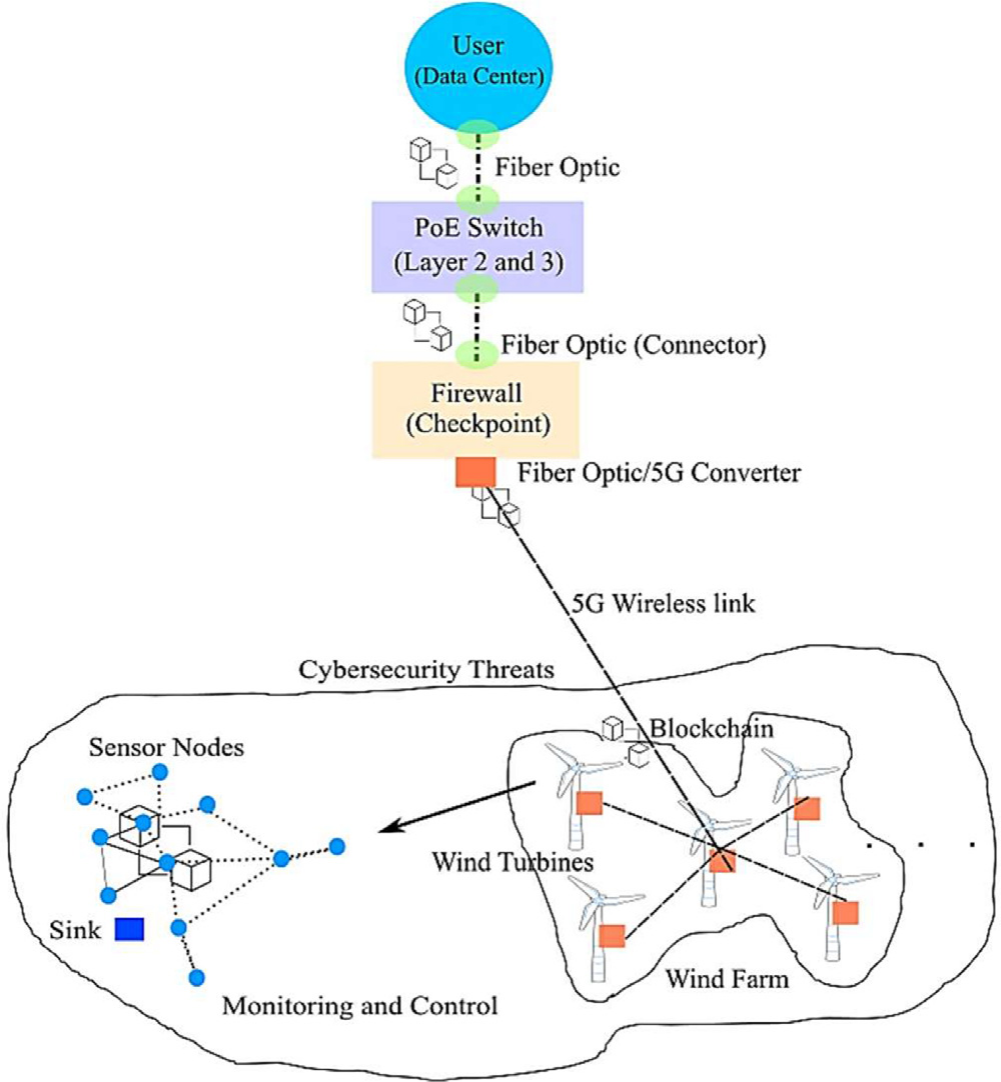
Wind-powered DERs in SG [1].

This paper presents datasets of Solana blockchain-based IWSNs deployed for the events monitoring and control in geographically distributed wind turbines in a wind farm. As part of the research methodology, real-world statistics on cyber events in Solana blockchain-based IWSNs in DERs are gathered and analyzed. These datasets contain details on different kinds of cyberattacks, their frequency, and the tactics used by attackers in energy and power systems. By examining these big datasets, researchers can identify common attack vectors, vulnerabilities, and potential weak points in the security framework of blockchain-based communication systems. For the sake of reusability, the measured cybersecurity datasets provided here are in .CSV (Comma Separated Values) format. As shown in Fig. 1, these datasets were collected and transmitted from the wind farm to the remote data center using hybrid (5G and Optical fiber) communication technologies, and stored in an MS SQL server in the SG. Statically deployed sensors were involved in computing and measuring various events such as, wind direction, speed, temperature, humidity, smoke, proximity, motion, cracks, current, voltage, frequency, etc.

During monitoring and control process, various cyberattacks including, SQL Injection, Spoofing, and Man-in-the-Middle were launched for data leakage, malicious tampering, and identity validity theft of the energy and power systems. The SQL Injection attack involves inserting malicious SQL code into a database query, allowing attackers to manipulate or steal data from the database. The Man-in-the-Middle attack allows an attacker to intercept and possibly alters the communication between two energy and power systems nodes without their knowledge, potentially manipulating the data being exchanged. On the other hand, in a spoofing attack, the attacker disguises themselves as a trusted entity to manipulate data, such as altering the information in the sensors and intelligent electronics devices cache or monitoring system website redirects.

**Fig. 2:**
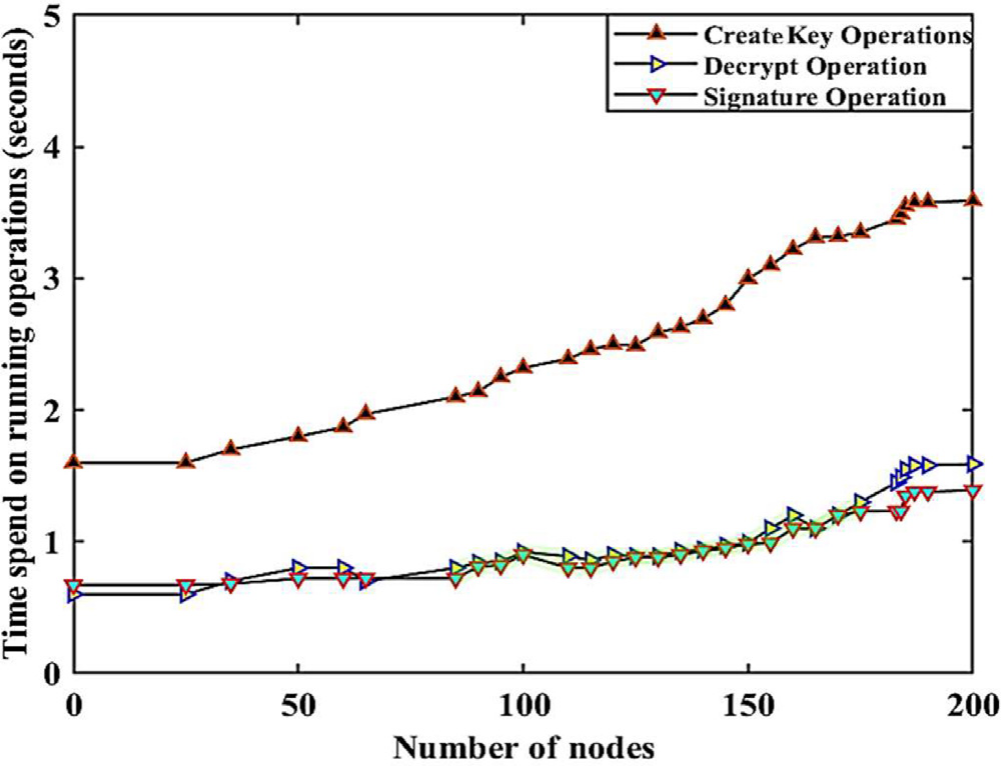
The relationship between the number of nodes and the time spent on running creating key, decryption, and signature operations in the smart grid.

**Fig. 3:**
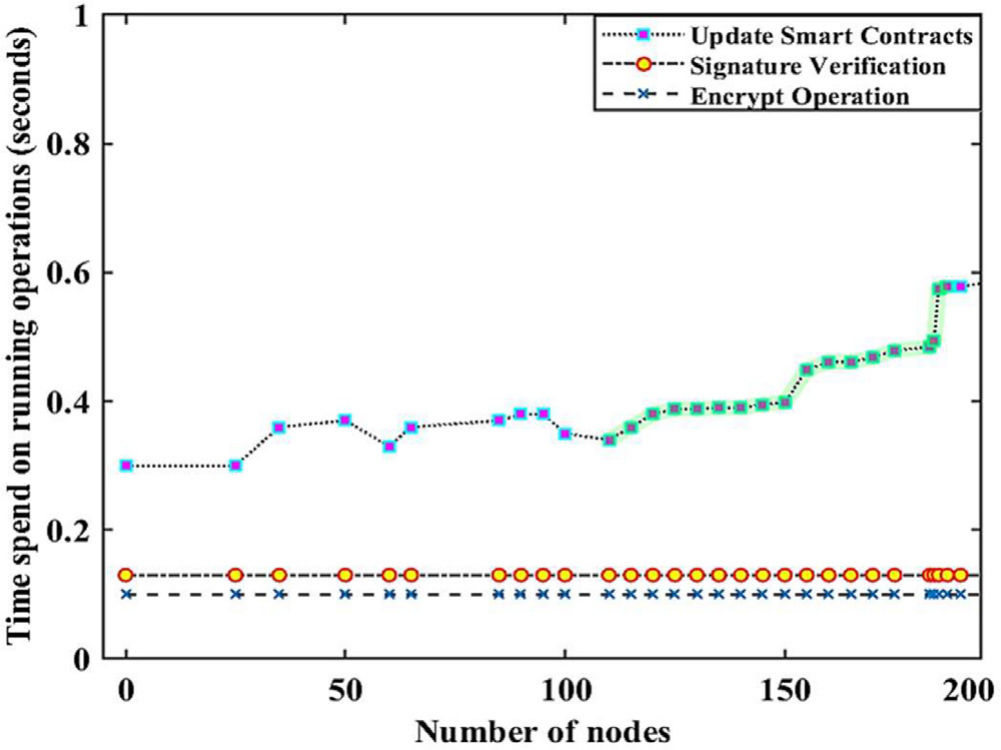
The relationship between the number of nodes and the time spent on updating smart contracts, signature verification, and encryption operations in the smart grid.

**Fig. 4:**
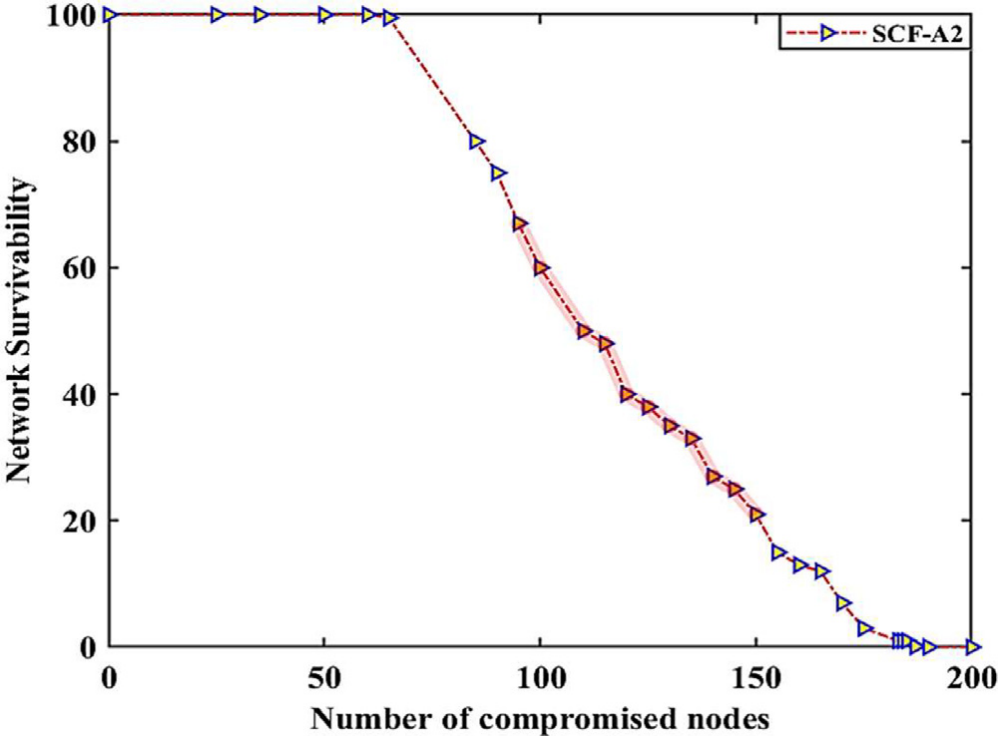
case (i), the relationship between the number of compromised nodes and the network resilience in the smart grid.

**Fig. 5:**
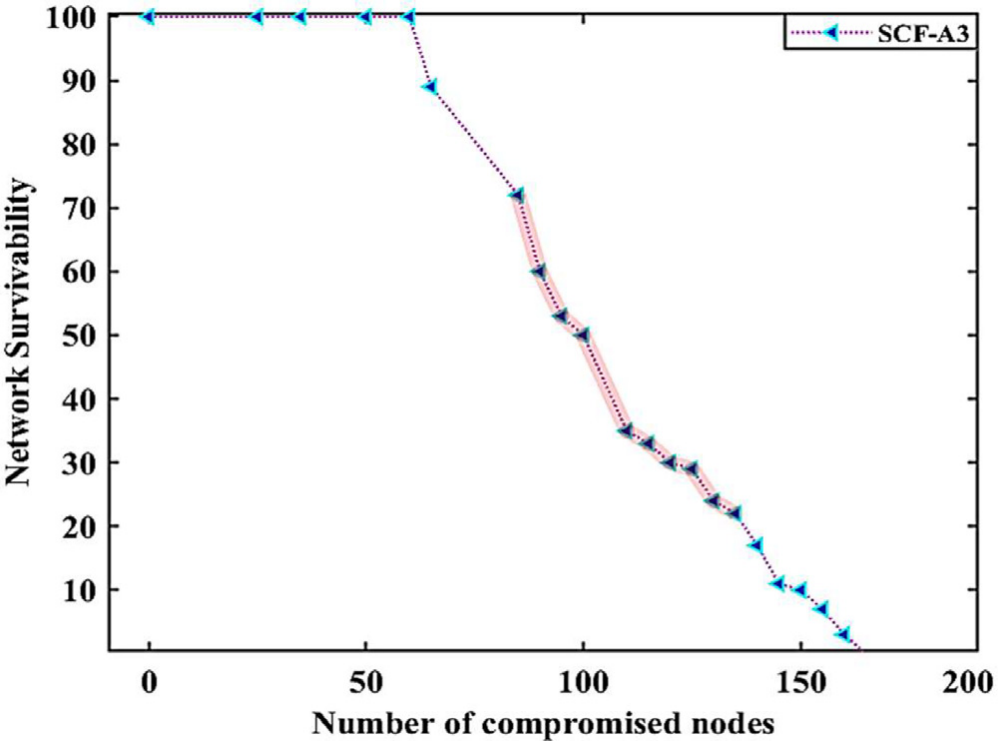
case (ii), the relationship between the number of compromised nodes and the network resilience in the smart grid.

**Fig. 6:**
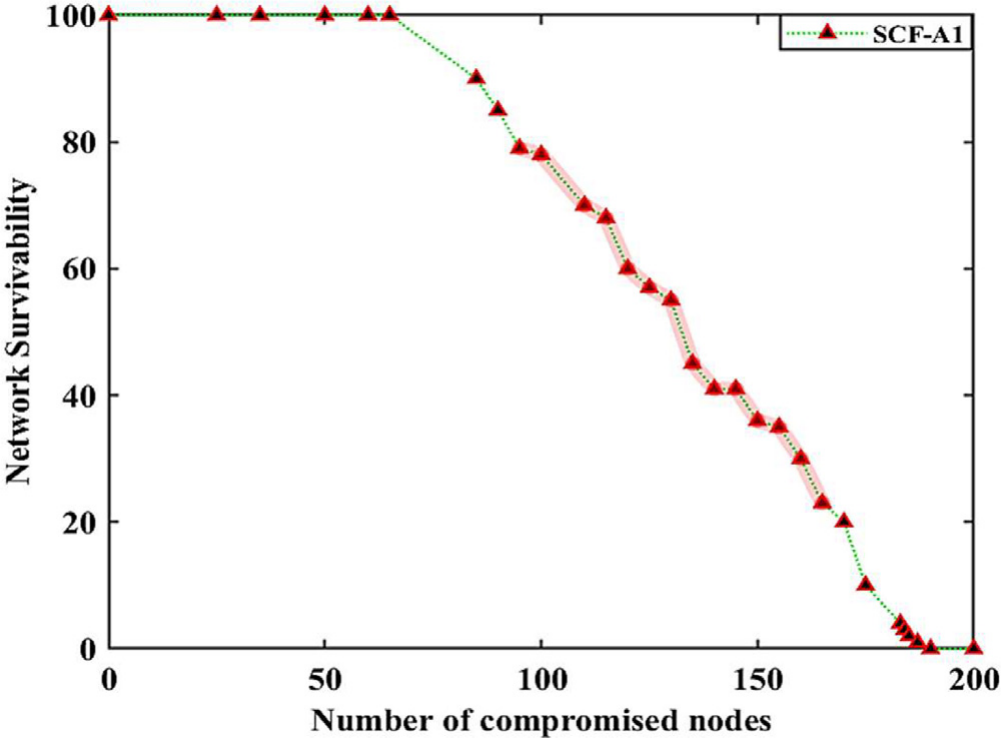
case (iii), the relationship between the number of compromised nodes and the network resilience in the smart grid.

In the simulation studies, 40 nodes (n) with their unique identity (e.g., node with unique identify number 1, is indicated as n1 and vice versa) were randomly selected to study the cyberattacks pattern in the smart grid. The frequency of measurements is configured to be real-time in intervals of every 30 min, and the values measured in the under-attack networks are given in Table 3, Table 4, Table 5, Table 6, Table 7, and their graphical representations are shown in Fig. 2, Fig. 3, Fig. 4, Fig. 5, Fig. 6. In addition, the values presented in tables (3 to 7) were converted from Megabits per second (Mbps) to Gigabits per second (Gbps) for a more clear understanding in the established network.

**Table 3:** Datasets for creating key, decryption, and signature operations in Solana blockchain-based IWSNs.

	Nodes	Latency in different operations		
No.	Metrics	CrK Avg. (≅)	DeC Avg. (≅)	SiG Avg. (≅)
1	n3	3.512380 ± 3.105263 2.512380 ± 2.329423 2.009519 ± 1.789845 1.103443 ± 0.030553 3.512543 ± 3.497473 1.909003 ± 1.141626 3.809850 ± 3.635323 2.344231 ± 1.213242 3.423441 ± 2.013142 2.009854 ± 1.083737 3.809857 ± 3.183737 1.657739 ± 0.711411 3.609800 ± 2.093838 3.985742 ± 3.326252 2.984750 ± 2.726210 3.029380 ± 0.003231 3.847298 ± 1.083737 1.243573 ± 0.031313 2.902309 ± 2.051525 2.290404 ± 2.003003 1.392374 ± 0.142320 1.203275 ± 1.152563 3.609388 ± 2.083737 2.109894 ± 1.162535 3.904584 ± 3.092772 1.905843 ± 1.000283 1.485753 ± 1.122201 2.607945 ± 2.025352	1.645353 ± 0.105263 1.563636 ± 0.329423 0.472620 ± 1.789845 1.028388 ± 0.020033 1.027366 ± 0.497473 1.635521 ± 0.141626 0.928730 ± 0.635323 0.424523 ± 0.213242 1.052202 ± 1.013142 0.002727 ± 0.001737 0.062553 ± 0.001343 0.987271 ± 0.772738 0.622022 ± 0.001200 1.700023 ± 1.923220 0.928820 ± 0.377200 1.052427 ± 0.827731 1.003520 ± 1.000021 1.100373 ± 1.007731 0.102883 ± 0.043325 0.290272 ± 0.092828 1.509234 ± 1.200491 0.354563 ± 0.226321 0.736634 ± 0.227370 1.735530 ± 1.364675 1.244353 ± 1.044536 1.530030 ± 0.983611 1.666353 ± 1.435532 1.635000 ± 1.637370 0.119324 ± 0.066572 1.613329 ± 1.355235 1.725421 ± 1.524419 1.400232 ± 1.005242 0.135352 ± 0.111731 1.377625 ± 1.203938 1.475764 ± 1.337228 1.283732 ± 1.100112 1.223234 ± 1.100391 1.577568 ± 1.036351 1.683773 ± 1.444390 1.538311 ± 1.500012	1.433400 ± 1.027372 1.300938 ± 1.112680 1.202823 ± 0.700923 1.111249 ± 0.882738 1.185354 ± 0.928281 1.322132 ± 0.726623 0.332858 ± 0.231108 0.982772 ± 0.724240 1.337262 ± 1.000232 1.103423 ± 0.992829 1.066501 ± 0.700271 1.127226 ± 1.000110 0.852423 ± 0.700281 1.238571 ± 1.100005 0.921121 ± 0.820020 1.211423 ± 0.988272 0.877364 ± 0.722021 0.277300 ± 0.011373 0.900927 ± 0.811026 1.262538 ± 1.092001 1.002821 ± 1.012214 1.027262 ± 0.827372 0.924421 ± 0.726266 0.735110 ± 0.711122 1.440015 ± 1.331104 1.023222 ± 0.936728 1.300277 ± 1.238283 1.011182 ± 1.000225 0.942332 ± 0.820390 0.862635 ± 0.069973 1.411001 ± 1.222320 1.402302 ± 1.226623 1.326621 ± 1.100061 0.988720 ± 0.827228 1.400021 ± 1.230224 0.982735 ± 0.930034 0.846293 ± 0.804758 1.211674 ± 1.002372 1.394846 ± 1.123243 0.788399 ± 0.657345
2	n5			
3	n9			
4	n13			
5	n14			
6	n17			
7	n20			
8	n29			
9	n33			
10	n39			
11	n50			
12	n62			
13	n68			
14	n71			
15	n75			
16	n79			
17	n81			
18	n84			
19	n88			
20	n91			
21	n97			
22	n105			
23	n109			
24	n119			
25	n120			
26	n128			
27	n138			
28	n143			
29	n144	0.849832 ± 1.093843		
30	n149	2.562536 ± 0.083282		
31	n159	1.102323 ± 1.160025		
32	n166	3.004522 ± 3.000232		
33	n169	0.905232 ± 0.023432		
34	n170	1.423422 ± 1.345433		
35	n183	2.343534 ± 2.234322		
36	n185	1.234353 ± 1.534232		
37	n189	3.232423 ± 2. 899,834		
38	n190	2.908303 ± 2.909843		
39	n192	3.075487 ± 3.267233		
40	n196	3.002224 ± 2.434222

**Table 4:** Datasets for updating smart contracts, signature verification, and encryption operations in Solana blockchain-based IWSNs.

	Nodes	Latency in different operations		
No.	Metrics	UsC Avg. (≅)	SiV Avg. (≅)	EnG Avg. (≅)
1	n3	1.322636 ± 0.904345 1.400223 ± 1.205767 1.444360 ± 1.387635 0.597834 ± 1.498767 0.923521 ± 0.882375 1.211011 ± 1.143521 1.355262 ± 1.157360 1.404245 ± 1.280012 1.367231 ± 1.281101 1.403292 ± 1.000352 1.228838 ± 1.002625 0.974739 ± 0.800151 0.811014 ± 0.708172 0.988371 ± 0.721010 1.401011 ± 1.201189 0.929380 ± 0.750194 0.823927 ± 0.645828 1.374663 ± 0.950911 1.395985 ± 1.356155 0.993737 ± 0.850291 0.924747 ± 0.830928 1.300200 ± 0.990021 1.329001 ± 1.060917 1.128273 ± 1.142316 1.102777 ± 0.929282 0.978423 ± 0.897254 0.880636 ± 0.742562 1.007883 ± 0.973020	0.130012 ± 0.096567 0.131121 ± 0.122754 0.123254 ± 0.982130 0.126546 ± 0.121030 0.119878 ± 0.095471 0.131212 ± 0.121812 0.122432 ± 0.109602 0.115345 ± 0.103000 0.131021 ± 0.113110 0.117634 ± 0.101498 0.118649 ± 0.099855 0.128753 ± 0.113903 0.126728 ± 0.109326 0.130171 ± 0.126544 0.126378 ± 0.117498 0.119564 ± 0.109900 0.119743 ± 0.109443 0.124610 ± 0.120001 0.108746 ± 0.093590 0.130010 ± 0.122025 0.119202 ± 0.100434 0.126863 ± 0.101330 0.130030 ± 0.120877 0.125302 ± 0.116435 0.134603 ± 0.134030 0.130731 ± 0.130004 0.129324 ± 0.119265 0.110007 ± 0.108775 0.114663 ± 0.106740 0.129800 ± 0.115000 0.120087 ± 0.118414 0.119034 ± 0.105254 0.131102 ± 0.121897 0.120340 ± 0.120030 0.125760 ± 0.107283 0.118730 ± 0.100517 0.130031 ± 0.110399 0.127875 ± 0.116398 0.130011 ± 0.129402 0.130300 ± 0.128566	0.100011 ± 0.099645 0.091765 ± 0.090806 0.100794 ± 0.993002 0.100023 ± 0.099263 0.093546 ± 0.092555 0.099556 ± 0.089925 0.098930 ± 0.097221 0.095434 ± 0.092887 0.101100 ± 0.989200 0.100231 ± 0.100001 0.096400 ± 0.094536 0.101244 ± 0.101010 0.093524 ± 0.093240 0.090765 ± 0.090010 0.094652 ± 0.094000 0.095436 ± 0.094438 0.100800 ± 0.098209 0.105305 ± 0.101111 0.094322 ± 0.093002 0.092111 ± 0.090181 0.093432 ± 0.091001 0.090121 ± 0.897718 0.100129 ± 0.099829 0.101200 ± 0.099827 0.097651 ± 0.092566 0.091322 ± 0.090025 0.102644 ± 0.089278 0.100073 ± 0.091750 0.100630 ± 0.100001 0.109027 ± 0.091879 0.090371 ± 0.091650 0.092852 ± 0.090108 0.102963 ± 0.091651 0.090031 ± 0.089167 0.093317 ± 0.091112 0.090001 ± 0.086091 0.090311 ± 0.090001 0.100878 ± 0.098990 0.095729 ± 0.090112 0.092628 ± 0.090011
2	n5			
3	n9			
4	n13			
5	n14			
6	n17			
7	n20			
8	n29			
9	n33			
10	n39			
11	n50			
12	n62			
13	n68			
14	n71			
15	n75			
16	n79			
17	n81			
18	n84			
19	n88			
20	n91			
21	n97			
22	n105			
23	n109			
24	n119			
25	n120			
26	n128			
27	n138			
28	n143			
29	n144	1.103485 ± 0.810228		
30	n149	0.827379 ± 0.659282		
31	n159	1.363566 ± 1.323141		
32	n166	0.977475 ± 0.965244		
33	n169	0.937374 ± 0.902313		
34	n170	0.884736 ± 0.802413		
35	n183	1.339228 ± 1.308915		
36	n185	1.335522 ± 0.927210		
37	n189	1.384774 ± 1.262416		
38	n190	1.432306 ± 1.208172		
39	n192	0.978540 ± 0.882619		
40	n196	1.146823 ± 1.091516

**Table 5:** Network resilience datasets when the network is attacked by SQL injection cyberattack in Solana blockchain-based IWSNs.

	Nodes	Network resilience operations in cyberattacks		
No.	Metrics	Normal data Avg. (≅)	Abnormal activity-5(a) Avg. (≅)	Abnormal activity-5(b) Avg. (≅)
1	n3	0.230383 ± 0.245409 0.210322 ± 0.201100 0.240871 ± 0.241091 0.207423 ± 0.208600 0.218520 ± 0.220254 0.211312 ± 0.213530 0.215226 ± 0.215768 0.204237 ± 0.208120 0.236211 ± 0.238111 0.203090 ± 0.201525 0.228002 ± 0.232254 0.214395 ± 0.212010 0.218144 ± 0.220007 0.219710 ± 0.221572 0.201615 ± 0.203126 0.229008 ± 0.231191 0.239208 ± 0.241805 0.204683 ± 0.201110 0.224980 ± 0.225613 0.239361 ± 0.243204 0.224401 ± 0.225920 0.230234 ± 0.231214 0.232021 ± 0.225091 0.225035 ± 0.226200 0.212706 ± 0.215823 0.207424 ± 0.209250 0.208366 ± 0.209562 0.201003 ± 0.202621	0.230383 ± 0.245409 0.210322 ± 0.201100 0.260132 ± 0.284145 0.207423 ± 0.208600 0.258554 ± 0.270000 0.211312 ± 0.213530 0.215226 ± 0.215768 0.204237 ± 0.208120 0.276111 ± 0.288000 0.100090 ± 0.101111 0.228002 ± 0.232254 0.214395 ± 0.212010 0.218144 ± 0.220007 0.019948 ± 0.021000 0.201274 ± 0.108400 0.329000 ± 0.301111 0.239208 ± 0.241805 0.200400 ± 0.100000 0.224980 ± 0.225613 0.239361 ± 0.243204 0.125841 ± 0.020090 0.230234 ± 0.231214 0.232021 ± 0.225091 0.225035 ± 0.226200 0.012950 ± 0.005003 0.207424 ± 0.209250 0.208366 ± 0.209562 0.201003 ± 0.202621 0.234820 ± 0.236102 0.014000 ± 0.259847 0.223506 ± 0.225417 0.267457 ± 0.278985 0.237247 ± 0.239013 0.208435 ± 0.210155 0.235220 ± 0.237159 0.205223 ± 0.207218 0.244001 ± 0.245413 0.001100 ± 0.000371 0.263001 ± 0.018111 0.224235 ± 0.225106	0.010111 ± 0.009000 0.210322 ± 0.201100 0.110171 ± 0.001011 0.207423 ± 0.208600 0.338000 ± 0.260040 0.211312 ± 0.213530 0.215426 ± 0.136642 0.204237 ± 0.208120 0.146374 ± 0.019930 0.001110 ± 0.101109 0.228002 ± 0.232254 0.214395 ± 0.212010 0.218144 ± 0.220007 0.119880 ± 0.227011 0.231155 ± 0.101603 0.094080 ± 0.019991 0.239208 ± 0.241805 0.004600 ± 0.001111 0.224980 ± 0.225613 0.239361 ± 0.243204 0.110081 ± 0.005410 0.050211 ± 0.051200 0.232021 ± 0.225091 0.225035 ± 0.226200 0.182003 ± 0.101522 0.104537 ± 0.204380 0.208366 ± 0.209562 0.034833 ± 0.034989 0.234820 ± 0.236102 0.290895 ± 0.249536 0.239236 ± 0.302343 0.310302 ± 0.329238 0.237247 ± 0.239013 0.208435 ± 0.210155 0.023243 ± 0.002345 0.205223 ± 0.207218 0.000122 ± 0.111100 0.018200 ± 0.012210 0.210011 ± 0.006823 0.224235 ± 0.225106
2	n5			
3	n9			
4	n13			
5	n14			
6	n17			
7	n20			
8	n29			
9	n33			
10	n39			
11	n50			
12	n62			
13	n68			
14	n71			
15	n75			
16	n79			
17	n81			
18	n84			
19	n88			
20	n91			
21	n97			
22	n105			
23	n109			
24	n119			
25	n120			
26	n128			
27	n138			
28	n143			
29	n144	0.234820 ± 0.236102		
30	n149	0.240300 ± 0.240982		
31	n159	0.223506 ± 0.225417		
32	n166	0.207510 ± 0.208245		
33	n169	0.237247 ± 0.239013		
34	n170	0.208435 ± 0.210155		
35	n183	0.235220 ± 0.237159		
36	n185	0.205223 ± 0.207218		
37	n189	0.244001 ± 0.245413		
38	n190	0.222300 ± 0.223170		
39	n192	0.218040 ± 0.218296		
40	n196	0.224235 ± 0.225106

**Table 6:** Network resilience datasets when the network is attacked by spoofing and man-in-the-middle cyberattacks in Solana blockchain-based IWSNs.

	Nodes	Network resilience operations in cyberattacks		
No.	Metrics	Normal data Avg. (≅)	Abnormal activity-6(a) Avg. (≅)	Abnormal activity-6(b) Avg. (≅)
1	n3	0.230383 ± 0.245409 0.210322 ± 0.201100 0.240871 ± 0.241091 0.207423 ± 0.208600 0.218520 ± 0.220254 0.211312 ± 0.213530 0.215226 ± 0.215768 0.204237 ± 0.208120 0.236211 ± 0.238111 0.203090 ± 0.201525 0.228002 ± 0.232254 0.214395 ± 0.212010 0.218144 ± 0.220007 0.219710 ± 0.221572 0.201615 ± 0.203126 0.229008 ± 0.231191 0.239208 ± 0.241805 0.204683 ± 0.201110 0.224980 ± 0.225613 0.239361 ± 0.243204 0.224401 ± 0.225920 0.230234 ± 0.231214 0.232021 ± 0.225091 0.225035 ± 0.226200 0.212706 ± 0.215823 0.207424 ± 0.209250 0.208366 ± 0.209562 0.201003 ± 0.202621	0.230383 ± 0.245409 0.210322 ± 0.201100 0.260132 ± 0.284145 0.207423 ± 0.208600 0.258554 ± 0.270000 0.0e1100 ± 0.2e3111 0.215226 ± 0.215768 0.204237 ± 0.208120 0.276111 ± 0.288000 0.100090 ± 0.101111 0.228002 ± 0.232254 0.1e0001 ± 0.1e0010 0.218144 ± 0.220007 0.019948 ± 0.021000 0.201274 ± 0.108400 0.329000 ± 0.301111 0.239208 ± 0.241805 0.200400 ± 0.100000 0.224980 ± 0.225613 0.239361 ± 0.243204 0.125841 ± 0.020090 0.230234 ± 0.231214 0.101091 ± 0.205001 0.225035 ± 0.226200 0.012950 ± 0.005003 0.207424 ± 0.209250 0.008356 ± 0.102534 0.201003 ± 0.202621 0.234820 ± 0.236102 0.014000 ± 0.259847 0.223506 ± 0.225417 0.267457 ± 0.278985 0.237247 ± 0.239013 0.108294 ± 0.218102 0.235220 ± 0.237159 0.205223 ± 0.207218 0.244001 ± 0.245413 0.001100 ± 0.000371 0.263001 ± 0.018111 0.224235 ± 0.225106	0.010111 ± 0.009000 0.210322 ± 0.201100 0.110171 ± 0.001011 0.207423 ± 0.208600 0.338000 ± 0.260040 0.1e1111 ± 0.1e3010 0.215426 ± 0.136642 0.204237 ± 0.208120 0.146374 ± 0.019930 0.001110 ± 0.101109 0.228002 ± 0.232254 0.1e1100 ± 0.110010 0.218144 ± 0.220007 0.119880 ± 0.227011 0.231155 ± 0.101603 0.094080 ± 0.019991 0.239208 ± 0.241805 0.004600 ± 0.001111 0.224980 ± 0.225613 0.239361 ± 0.243204 0.110081 ± 0.005410 0.050211 ± 0.051200 0.0e0251 ± 0.1e1001 0.225035 ± 0.226200 0.182003 ± 0.101522 0.104537 ± 0.204380 0.1e0112 ± 0.001412 0.034833 ± 0.034989 0.234820 ± 0.236102 0.290895 ± 0.249536 0.239236 ± 0.302343 0.310302 ± 0.329238 0.237247 ± 0.239013 0.1e0301 ± 0.110101 0.023243 ± 0.002345 0.205223 ± 0.207218 0.000122 ± 0.111100 0.018200 ± 0.012210 0.210011 ± 0.006823 0.224235 ± 0.225106
2	n5			
3	n9			
4	n13			
5	n14			
6	n17			
7	n20			
8	n29			
9	n33			
10	n39			
11	n50			
12	n62			
13	n68			
14	n71			
15	n75			
16	n79			
17	n81			
18	n84			
19	n88			
20	n91			
21	n97			
22	n105			
23	n109			
24	n119			
25	n120			
26	n128			
27	n138			
28	n143			
29	n144	0.234820 ± 0.236102		
30	n149	0.240300 ± 0.240982		
31	n159	0.223506 ± 0.225417		
32	n166	0.207510 ± 0.208245		
33	n169	0.237247 ± 0.239013		
34	n170	0.208435 ± 0.210155		
35	n183	0.235220 ± 0.237159		
36	n185	0.205223 ± 0.207218		
37	n189	0.244001 ± 0.245413		
38	n190	0.222300 ± 0.223170		
39	n192	0.218040 ± 0.218296		
40	n196	0.224235 ± 0.225106

**Table 7:** Network resilience datasets when the network is attacked by SQL injection, spoofing, and man-in-the-middle cyberattacks in Solana blockchain-based IWSNs.

	Nodes	Network resilience operations in cyberattacks		
No.	Metrics	Normal data Avg. (≅)	Abnormal activity-7(a) Avg. (≅)	Abnormal activity-7(b) Avg. (≅)
1	n3	0.230383 ± 0.245409 0.210322 ± 0.201100 0.240871 ± 0.241091 0.207423 ± 0.208600 0.218520 ± 0.220254 0.211312 ± 0.213530 0.215226 ± 0.215768 0.204237 ± 0.208120 0.236211 ± 0.238111 0.203090 ± 0.201525 0.228002 ± 0.232254 0.214395 ± 0.212010 0.218144 ± 0.220007 0.219710 ± 0.221572 0.201615 ± 0.203126 0.229008 ± 0.231191 0.239208 ± 0.241805 0.204683 ± 0.201110 0.224980 ± 0.225613 0.239361 ± 0.243204 0.224401 ± 0.225920 0.230234 ± 0.231214 0.232021 ± 0.225091 0.225035 ± 0.226200 0.212706 ± 0.215823 0.207424 ± 0.209250 0.208366 ± 0.209562 0.201003 ± 0.202621	0.230383 ± 0.245409 0.1027948 ± 0.201100 0.260132 ± 0.284145 0.210001 ± 0.218011 0.258554 ± 0.270000 0.0e1100 ± 0.2e3111 0.210284 ± 0.222098 0.000937 ± 0.000120 0.276111 ± 0.288000 0.100090 ± 0.101111 0.008001 ± 0.006252 0.1e0001 ± 0.1e0010 0.018011 ± 0.034775 0.019948 ± 0.021000 0.201274 ± 0.108400 0.329000 ± 0.301111 0.000259 ± 0.002802 0.200400 ± 0.100000 0.0e0080 ± 0.e01130 0.239361 ± 0.243204 0.125841 ± 0.020090 0.230234 ± 0.231214 0.101091 ± 0.205001 0.009039 ± 0.132000 0.012950 ± 0.005003 0.207424 ± 0.209250 0.008356 ± 0.102534 0.201003 ± 0.202621 0.238909 ± 0.006112 0.014000 ± 0.259847 0.223506 ± 0.225417 0.267457 ± 0.278985 0.237247 ± 0.239013 0.108294 ± 0.218102 0.235220 ± 0.237159 0.23e348 ± 0.e00002 0.244001 ± 0.245413 0.001100 ± 0.000371 0.263001 ± 0.018111 0.004223 ± 0.e52340	0.010111 ± 0.009000 0.*e*× 02 × 0 ± 0.× 01,341 0.110171 ± 0.001011 0.e0 ×× 23 ± 0.10 × 20 × 0.338000 ± 0.260040 0.1e1111 ± 0.1e3010 0.215426 ± 0.136642 0.e2431×± 0.31 × 120 0.146374 ± 0.019930 0.001110 ± 0.101109 0.020002 ± 0.01225 × 0.1e1100 ± 0.110010 0.e0014 ×± 0.e10 × 09 0.119880 ± 0.227011 0.231155 ± 0.101603 0.094080 ± 0.019991 0.e29 × 5 ×± 0.2 × 6800 0.004600 ± 0.001111 0.0 × 5260 ± 0.24 × 644 0.239361 ± 0.243204 0.110081 ± 0.005410 0.050211 ± 0.051200 0.0e0251 ± 0.1e1001 0.10 × 035 ± 0.1 × 620 × 0.182003 ± 0.101522 0.104537 ± 0.204380 0.1e0112 ± 0.001412 0.034833 ± 0.034989 0.e30 ×× 0 ± 0.× 06,102 0.290895 ± 0.249536 0.239236 ± 0.302343 0.310302 ± 0.329238 0.237247 ± 0.239013 0.1e0301 ± 0.110101 0.023243 ± 0.002345 0.0052 ××± 0.207 ×× 8 0.000122 ± 0.111100 0.018200 ± 0.012210 0.210011 ± 0.006823 0.104 × 03 ± 0.1 ×× 109
2	n5			
3	n9			
4	n13			
5	n14			
6	n17			
7	n20			
8	n29			
9	n33			
10	n39			
11	n50			
12	n62			
13	n68			
14	n71			
15	n75			
16	n79			
17	n81			
18	n84			
19	n88			
20	n91			
21	n97			
22	n105			
23	n109			
24	n119			
25	n120			
26	n128			
27	n138			
28	n143			
29	n144	0.234820 ± 0.236102		
30	n149	0.240300 ± 0.240982		
31	n159	0.223506 ± 0.225417		
32	n166	0.207510 ± 0.208245		
33	n169	0.237247 ± 0.239013		
34	n170	0.208435 ± 0.210155		
35	n183	0.235220 ± 0.237159		
36	n185	0.205223 ± 0.207218		
37	n189	0.244001 ± 0.245413		
38	n190	0.222300 ± 0.223170		
39	n192	0.218040 ± 0.218296		
40	n196	0.224235 ± 0.225106

Table 3 illustrates the datasets for creating key (CrK), decryption (DeC), and signature (SiG) operations in the Solana blockchain-based IWSNs. It can be seen that the maximum and minimum latency values of CrK are changing between 3.80 and 0.03 for the randomly selected nodes in the SG. The high and low latency values of DeC are observed between 1.74 and 0.0013 for the randomly selected nodes in the SG. In addition, the maximum and minimum latency values of SiG for the randomly selected nodes are observed between 1.44 and 0.01 in the smart grid. The data presented in Table 3 highlights that the CrK latency value is higher compared to both DeC and SiG in the SG. On the other hand, the DeC latency value is slightly higher than the SiG, and most of the time both latency values overlap each other, as shown in Fig. 2.

Table 4 presents the datasets for updating smart contracts (UsC), signature verification (SiV), and encryption (EnG) operations in the Solana blockchain-based IWSNs. It is observed that the high and low latency values of UsC are changing between 1.44 and 0.64 for the randomly selected nodes in the SG. On the other hand, the maximum and minimum latency values of SiV and EnG are changing between 134 and 0.093, and 0.1053 and 0.086, respectively. The data presented in Table 4 clearly shows that the EnG latency value is low compared to both UsC and SiV in the SG. Most of the time, the EnG and SiV latency values overlap each other in the SG. The latency value of UsC is recorded high compared to both SiV and EnG as highlighted in Fig. 3.

Case (i): Table 5 indicates the network resilience datasets when the nodes are involved in malicious activity in case of single type of SQL Injection cyberattack, introduced by the adversary in the Solana blockchain-based IWSNs. The first column in Table 5 shows the normal data shared between different nodes during events monitoring and control in the DERs. On the other hand, malicious activity between specific nodes in the data-sharing process in the network is shown in columns 5(a) and 5(b), respectively. The highlighted datasets in columns 5(a) and 5(b) represent the facts when 50% and 70% of the nodes in the network are involved in malicious activities in the DERs. The highlighted datasets in these columns express that the value of data is changing frequently in the case of a single kind of cyberattack in the network. After analyzing the datasets of the randomly selected specific nodes having unique identities, e.g., n9 and n39, it is noticed that the data shared between nodes over a communication link is higher than the data packets generated in the network. On the other hand, it is also found that the data shared between nodes over a communication link is extremely low compared to the data packets generated in the network. Such types of cyberattacks may lead to memory overflow and invalid data packet issues in the Solana blockchain-based IWSNs. The impact of network resilience against a single type of attack is shown in Fig. 4.

Case (ii): Table 6 highlights the network resilience datasets when the nodes are involved in malicious activity in case of multiple cyberattacks ≤2 (Spoofing and Man-in-the-Middle), introduced by the adversary in the Solana blockchain-based IWSNs. The first column in Table 6 shows the normal data shared between nodes during events monitoring and control, while the highlighted datasets in columns 6(a) and 6(b) illustrate when 60% and 80% of the nodes in the network are involved in malicious activities in the SG. The highlighted columns show the frequent change in datasets value when the nodes are involved in malicious activities under multiple cyberattacks in the DERs. In such cases, we notice several malicious activities of the nodes, including (i) bulk data packets being shared between nodes to create memory overflow and bandwidth utilization issues (ii) invalid data packets being shared between nodes to create systems monitoring and control issues, and (iii) empty data packets were routed between the nodes to enlarge overheads in the network. These observations are made by considering the malicious activities of the specific nodes having the unique identities, e.g., n14, n62, n84, n170, etc. The impact of network resilience against multiple cyberattacks is shown in Fig. 5.

Case (iii): Table 7 highlights the network resilience datasets when the nodes are involved in malicious activity in case of multiple cyberattacks >2 and ≤5 multiple (SQL Injection, Spoofing, and Man-in-the-Middle), launched by the adversary in the Solana blockchain-based IWSNs. In Table 7, the highlighted datasets in columns 7(a) and 7(b) illustrate when 80% and 95% of the nodes in the network are involved in malicious activities in the SG. In case of multiple cyberattacks, we noticed several malicious activities of the nodes, including the aforementioned (i) data packets embedded with misleading information being shared between the nodes for misleading control of the power generation and distribution systems, and (ii) data packets with missing information being shared between the nodes to lose control of the smart grid. These observations were made by considering the malicious activities of the specific nodes having the unique identities, e.g., n14, n62, n84, n170, n196, etc. The impact of network resilience against multiple cyberattacks is shown in Fig. 6.

## 4 Experimental Design, Materials, and Methods

In this study, a virtual machine Fedora32 installed on a local server with programming tools Metaplex and Rust is used to simulate the blockchain architecture in combination with RTDS/OPAL-RT in the smart grid. In the wind farm, each wind turbine was equipped with at least 9 multifunction sensors for temperature, humidity, smoke, proximity, motion, cracks, current, and voltage measurements in the energy and power systems. The path loss model [23] is used to simulate a point-to-point communication environment in each wind turbine located in different regions in the SGs. In addition, the positioning method [24] is employed to find the appropriate location of each node in the system along with perfect synchronization between power equipment and nodes in the Solana blockchain-based IWSNs [25]. In addition, the missing or manipulated data values of a sensor node $SN_{i}$ involved in events monitoring were obtained using neighboring nodes matrix technique in which the average data flow $D_{f}\left(SN_{i}\right)$ of the neighboring nodes $SN_{j}$ is observed in an event region k in time $t_{i}$ in the SG. This can be numerically illustrated as

(1) $$SN_{i}=\underset{j=1\to n}{Avg}D_{f}\left(SN_{i}\right)\sum {\left(SN_{j}\right)}^{k}t_{i}\;$$

## Limitations

There are some limitations with the datasets. First, the extent and variety of the datasets may not adequately cover all types of stealthy cyberattack scenarios, particularly the new ones. Therefore, it would be advantageous to generate synthetic datasets using machine learning techniques and integrate with the given datasets to encompass a broader spectrum of attack vectors and novel forms of cyberthreats in various energy and power system applications. Second, because the cybersecurity landscape is changing quickly, it is possible that the datasets may not be sufficient to adequately represent all types of network setups and user habits in diverse cyberattacks environments in smart grid. Therefore, enhancing the datasets to encompass a wider range of real-world network infrastructures might further improve the blockchain-based communication networks for power generation, transmission, and distribution systems. In future studies, the researchers might explore these issues to address cybersecurity challenges in a large-scale distributed energy and power systems.

## CRediT authorship contribution statement

**Muhammad Faheem:** Writing – original draft, Conceptualization, Methodology, Software, Validation. **Mahmoud Ahmad Al-Khasawneh:** Methodology, Software, Data curation. **Arfat Ahmad Khan:** Data curation. **Syed Hamid Hussain Madni:** Investigation, Validation.

## Data Availability

Cyberattacks Patterns in Blockchain-Based Communication Networks for Distributed Renewable Energy Systems: A study on datasets (Original data) (Mendeley Data).
